# Self-organization to sub-criticality

**DOI:** 10.1186/1471-2202-16-S1-O19

**Published:** 2015-12-18

**Authors:** V Priesemann

**Affiliations:** 1 Department of Nonlinear Dynamics, Max Planck Institute for Dynamics and Self-Organization, Göttingen, Germany; 2 Bernstein Center for Computational Neuroscience, Göttingen, Germany

## 

Human brains possess sophisticated information processing capabilities, which rely on the interactions of billions of neurons. However, it is unclear how these capabilities arise from the collective spiking dynamics. A popular hypothesis is that neural networks assume a critical state [1,2], because in models criticality maximizes information processing capabilities [3,4]. However, it has been largely overlooked that criticality in neural networks also comes with the risk of spontaneous runaway activity [5], which has been linked to epilepsy. Does the brain indeed assume a critical state, despite the risk of instability? To revisit this question, we analyzed spiking activity from awake animals, instead of more coarse measures of neural activity (population spikes, LPF, EEG, BOLD) as in most previous studies. In all recordings (rats hippocampus, cats visual cortex, and monkey prefrontal cortex), spiking activity resembled a sub-critical state, not criticality proper [6]. We confirmed these results using a novel mathematical approach that is robust to subsampling effects [7] [see Wilting & Priesemann, conference proceedings CNS 2015]. While 'self-organization' to criticality has been widely studied (e.g.[5,8]), it is unclear what mechanism allows self-organize to sub-criticality instead. Here, we demonstrate that homeostatic plasticity [9] assures that networks assume a slightly sub-critical state, independently of the initial configuration. Surprisingly, increasing the external input (stimuli) altered the set-point of the network to a more sub-critical state. Our results suggest that homeostasis allows the brain to maintain a safety margin to criticality. Thereby the brain may lose processing capability, but avoids instability.
